# The rapid synthesis of 1,10-phenanthroline-5,6-diimine (Phendiimine) and its fascinating photo-stimulated behavior

**DOI:** 10.1038/s41598-024-59272-4

**Published:** 2024-04-11

**Authors:** Ghasem Marandi, Ali Hassanzadeh

**Affiliations:** 1 Department of Organic Chemistry, Faculty of Chemistry, Urmia University, Urmia, Iran; 2 Department of Physical Chemistry, Faculty of Chemistry, Urmia University, Urmia, Iran

**Keywords:** 1,10-Phenanthroline-5,6-diimine (Phendiimine), 1,10-Phenanthroline-5,6-dione, Photoluminescence, Electrochemical behavior, Light dependent resistance, Optics and photonics, Materials chemistry, Organic chemistry, Photochemistry

## Abstract

Here, for the first time, we report synthesis of 1,10-phenanthroline-5,6-diimine (Phendiimine) based on an acid catalysed SN_2_ reaction of 1,10-phenanthroline-5,6-dione and 2-picolylamine in EtOH as a solvent. The synthesized Phendiimine molecule showed excellent photo-sensitivity against visible light, together with photoluminescence in both water and ethanol and also, it showed electrochemical activity with Fe electrode in ethanol and H_2_SO_4_ solution. Tauc plot also showed Phendiimine is a direct band-gap semiconductor. The hot-point probe test also showed that it is a n-type semiconductor. The UV–vis. absorption maximum shift in two solvents (water and ethanol) demonstrates the solvatochromism behavior of the molecule. The practical significance of this work and its guiding implication for future related research can be outlined as follows. Based on the results obtained, it appears that the Phendiimine molecule could revolutionize the medical field, potentially in the design of artificial eyes, increasing the yield of photovoltaic cells through enhanced heat transfer, improving computers and industrial photo-cooling systems, serving as photo-controller in place of piezoelectric devices, functioning as electronic opt couplers, controlling remote lasers, changing convection in photothermal heaters, designing miniaturized real photo-stimulated motors, creating photo or thermal switches through spin crossover complexes, developing electronic light-dependent resistance (LDR) devices, constructing X-ray and gamma-ray detectors, designing intelligent clothing, creating photo dynamic tumour therapy (PDT) complexes, singlet fission materials in solar cells and more.

## Introduction

One of the interesting phenomenon in chemistry world is Brownian motion of molecules^1–3^. When the small particles suspended in a liquid or a gas as a fluid, their collision results a random movement of this particles^4^. The understanding of such dynamic behaviors can be interesting and essential in the industrial aspects. Moreover, movements that are only horizontal or vertical in a specific direction can be very fascinating and remarkable^5^. There are some measurement methods and tools to develop gained information about the dynamic behavior rather a flow^6^. For example, infrared cameras and thermo-chromic liquid crystals. Some of these methods require very complex and expensive tools to observe these types of flows^7,8^. One of the important organic compounds with high photo active properties is 1,10-phenantroline^9–12^. This molecule and its derivatives show distinct properties in chemistry fields such as coordination chemistry, medicinal chemistry, industrial chemistry, etc^13–21^.

Based on our knowledge from literatures; 1,10-Phenanthroline-5,6-diimine (Phendiimine) is an organic molecule which has not been yet directly synthesized and there isn’t any recipe for its synthesis in literatures. But, it’s made indirectly from phendiamine oxidation using Ruthenium salt and air as reported by Fletcher et al.

In continuation of our previous works^22–25^, we report a facile and direct synthesis recipe for Phendiimine generation.

Furthermore, in this context, we observed wonderful behaviors of Phendiimine molecule in different solvents regarding its solvatochromism, photo-sensitivity or light dependent resistance (LDR), together with photoluminescence and electrochemical redox activity^26^.

A density functional theory (DFT) based study has been reported for Fe and Co complexation with 1,10-Phenanthroline-5,6-Diimine by Starikova et al. for using them as spin crossover complexes^27^. Recently, a paper regarding Ruthenium complex with phendione and Phendiimine oxidation has also been reported by Ghumaan et al. in which Phendiimine based complex has exhibited a better performance than the phendione based complex with ruthenium^28^.

**Figure 1:**
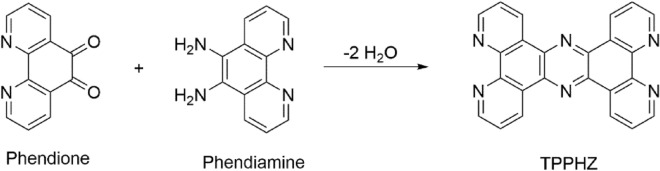
Reaction between phendione and phendiamine molecules to produce TPPHZ.

The main reason for less attention has been paid to direct synthesis of Phendiimine molecule is that it yields a mixture of phendiamine, Phenmonoimine, Phendiimine, and other side reactions between reactants and products which yields such as tetrapyrido [3,2-*a*:2′,3′-*c*:3′′,2′′-*h*:2′′′,3′′′-*j*] phenazine (TPPHZ) molecule and so on which has been shown in Fig. 1^14,16,29,30^.

## Results and discussion

### Synthesis and characterization

**Figure 2:**
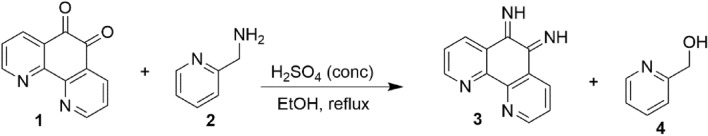
Synthesis of 1,10-phenanthroline-5,6-diimine.

The reaction between 1,10-phenanthroline-5,6-dione and 2-Picolylamin in the presence of concentrated H_2_SO_4_ as a homogeneous catalyst in ethanol as solvent led to 1,10-phenanthroline-5,6-diimine (Phendiimine) in excellent yields (Fig. 2).

Compound **3** has stable structure that spectroscopic analysis confirms its structure. The FT-IR spectrum of compound **3** shows N–H and C = N peaks at 3440 and 1570 cm^−1^, respectively. The ^1^H-NMR spectrum of compound 3 exhibits four distinct signals which is attributed to aromatic and imine protons. A doublet of doublet signal at *δ* = 7.15 with *J* = 8 and 4 Hz, a doublet at *δ* = 8.57 (*J* = 8 Hz) and another doublet at *δ* = 8.00 (*J* = 4 Hz). The proton of imine resonates at *δ* = 9.47 as a broad singlet.

**Figure 3:**
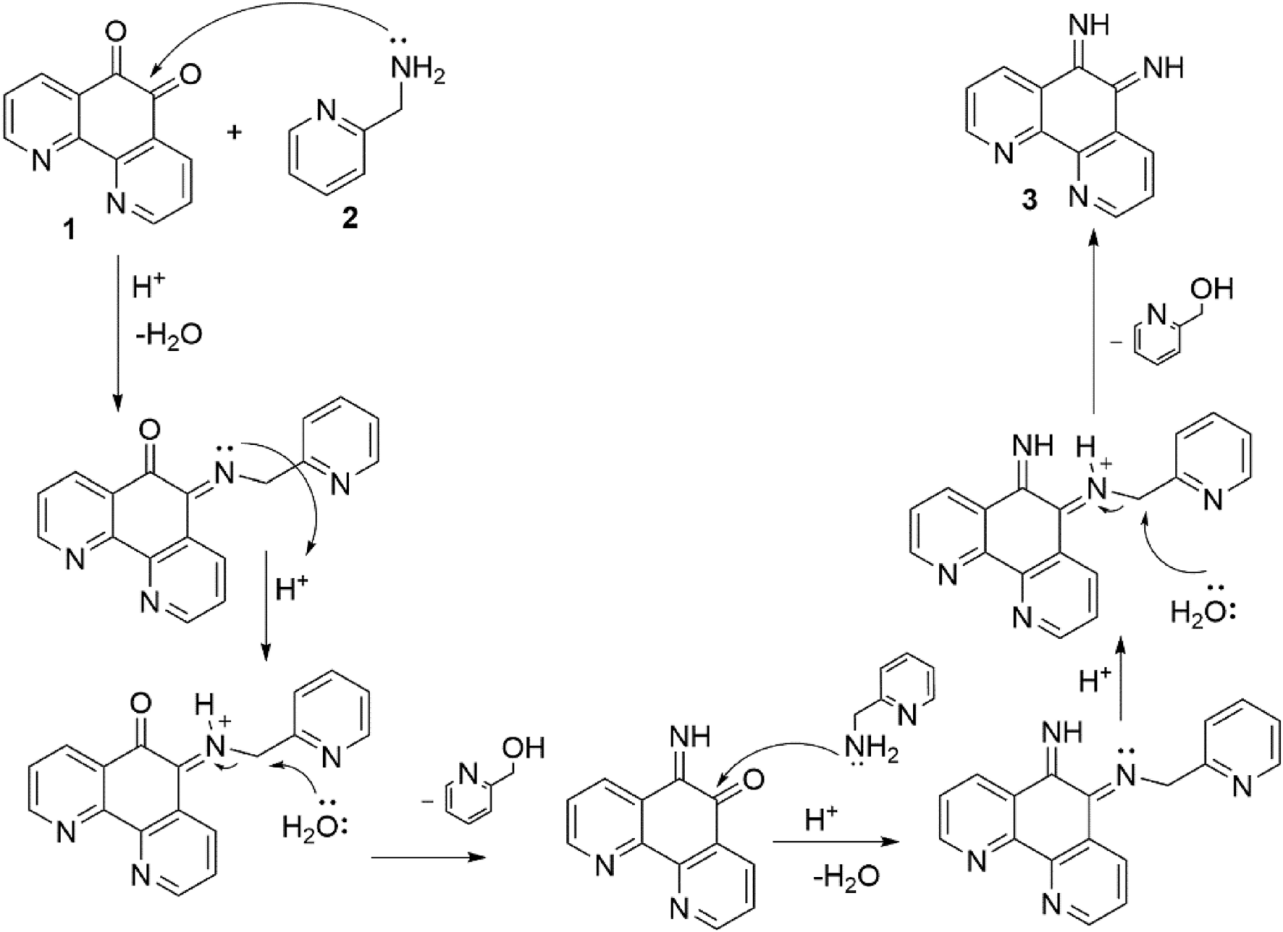
Illustrative mechanism of 1,10-phenanthroline-5,6-diimine (Phendiimine) synthesis.

An illustrative mechanism for the generation of compound **3** has been shown in Fig. 3. As can be seen, the reaction proceeds with the production of iminium ion, and then the active iminium ion is converted to the corresponding imine due to the attack of H_2_O molecules through a SN_2_ reaction.

### Photo-sensitivity against visible light

**Figure 4:**
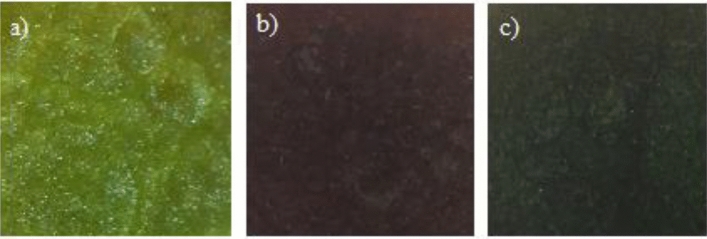
The colour of solution of compound **3** in ethanol: (**a**) before removal of solvent, (**b**) one year after synthesis by adding solvent, (**c**) after adding of 5 mL of diethyl ether to solution of compound **3**.

Compound **3** showed an interesting phenomenon, that after cooling the reaction mixture, there were still particles inside the hookah solution, and this continued until the moment when the ethanol solvent was present, and its colour was light green (Fig. 4, part a). The reaction solvent was removed by slow evaporation and the precipitate was washed with cold ether and the remaining solids were dried. After one year, ethanol was added to the solvent solids and it was observed that the particles inside the solution started to move again and the colour of the solution became brown (Fig. 4, part b). Then, by adding a small amount of ether to the same solution, the colour changed again and appeared in a rich green colour, but the movement of the particles inside the solution still continued (Fig. 4, part c).

**Figure 5:**
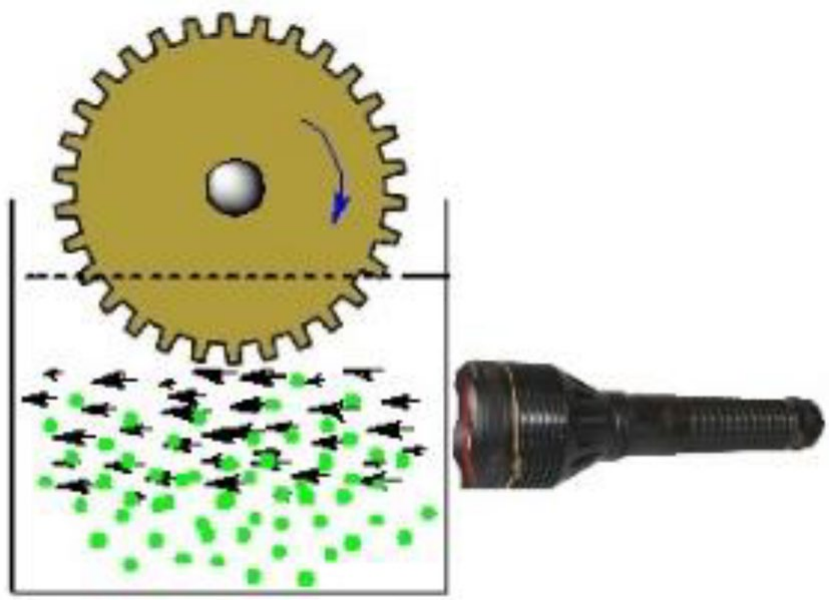
The movement of Phendiimine in the irradiated light direction in ethanol.

Since the Nobel Prize in chemistry was awarded to Ben L. Feringa and his co-workers in 2016, molecular motors have received a great deal of attention from scientists. This type of molecules is an important class of molecular machines. Distinct properties of these molecules from macroscopic machines are working in solution systems.^1,31–33^ These motors can run as long as the source of excitation (viz. light) is available. The synthesized Phendiimine molecule also has this property and authors observed that in the experiments; this molecule in ethanol as solvent can move forward in the path of the irradiated light and push the particles forward (see Fig. 5).

Furthermore, in this context, authors faced with many problems in determining Phendiimine molecules rotational speed and gaining further insight. On one hand, molecular motors have a rotational speed ranging from micro RPM to Mega RPM^33–35^. On the other hand, traditional tachometers can only measure rotational speeds from 0.5 RPM (with contact) to 100,000 RPM (contactless), and the human eye can only perceive phenomena with rotational speed lower than 60 Hz (approximately 3600 RPM). However, the apparent rotational movement of some Phendiimine molecules in a solvent can be seen with the naked eye, indicating a rotational speed of about 3000 RPM. Unfortunately, the authors have not yet found a suitable technique, other than capturing a video of its movement, to determine this low rotational speed of Phendiimine molecules under visible light irradiation.

### Photo-absorption study

**Figure 6:**
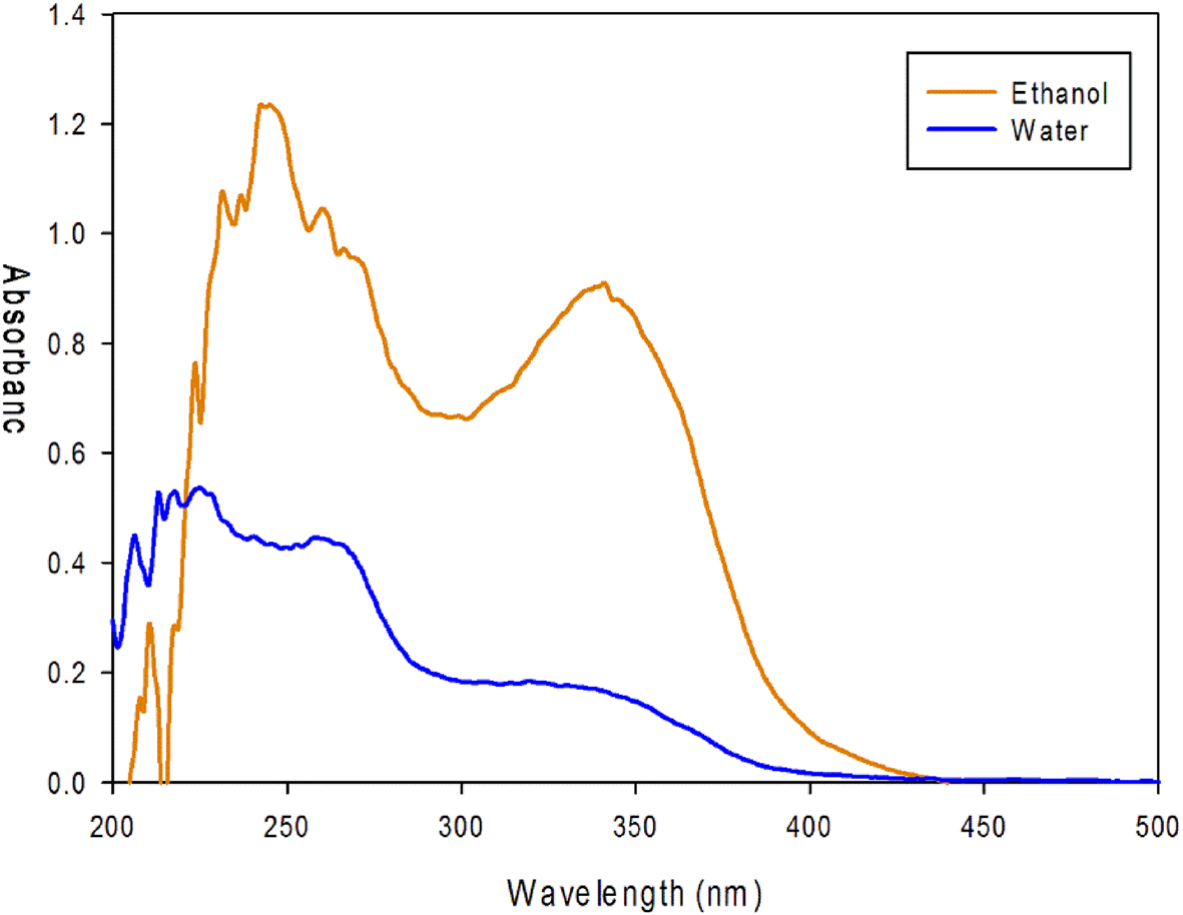
UV–Visible spectra of Phendiimine molecule in ethanol and water solvents.

Figure 6 shows comparative UV–visible spectra of Phendiimine molecule in ethanol and water as solvent. As can be seen that the maximum band in the range of 300–400 nm has been shifted to longer wavelengths (as a 22 nm red shift) in ethanol solvent. It means that these transitions have been done in ethanol easier than water solvent and with higher probability. The band is red-shifted, suggesting the involvement of the nitrogen atom lone pair in electron delocalization. This behavior is consistent with the ground-state destabilization of the Phendiimine molecule′s permanent dipole by the solvent. Spectral shifts in UV–Visible spectroscopy can result from various factors, including changes in the electronic structure of the molecule, alterations in the molecular environment, and instrumental factors. The red shift depends on the solvent’s polarizability, the oscillator strength (probability of absorption or emission) of the transition, and some of the higher energy transitions. Transitions in the 200–300 nm range belong to π → π* transitions, while those in the 300–450 nm range belong to n → π* transitions. Because the absorbance of the Phendiimine molecule falls within the UV and somewhat violet region of the electromagnetic spectrum, the compound appears yellow to the human eye. The absorption maximum shift in two solvents (water and ethanol) demonstrates the solvatochromic behavior of the Phendiimine molecule.

### Photo-luminescence study

**Figure 7:**
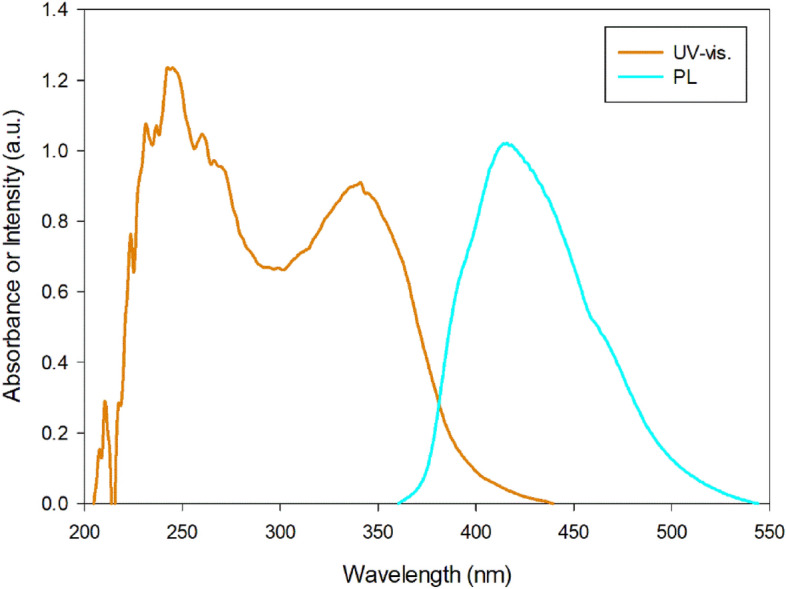
Photo-luminescence spectra of Phendiimine molecule in ethanol solvent with excitation wavelength = 200 nm at room temperature.

**Figure 8:**
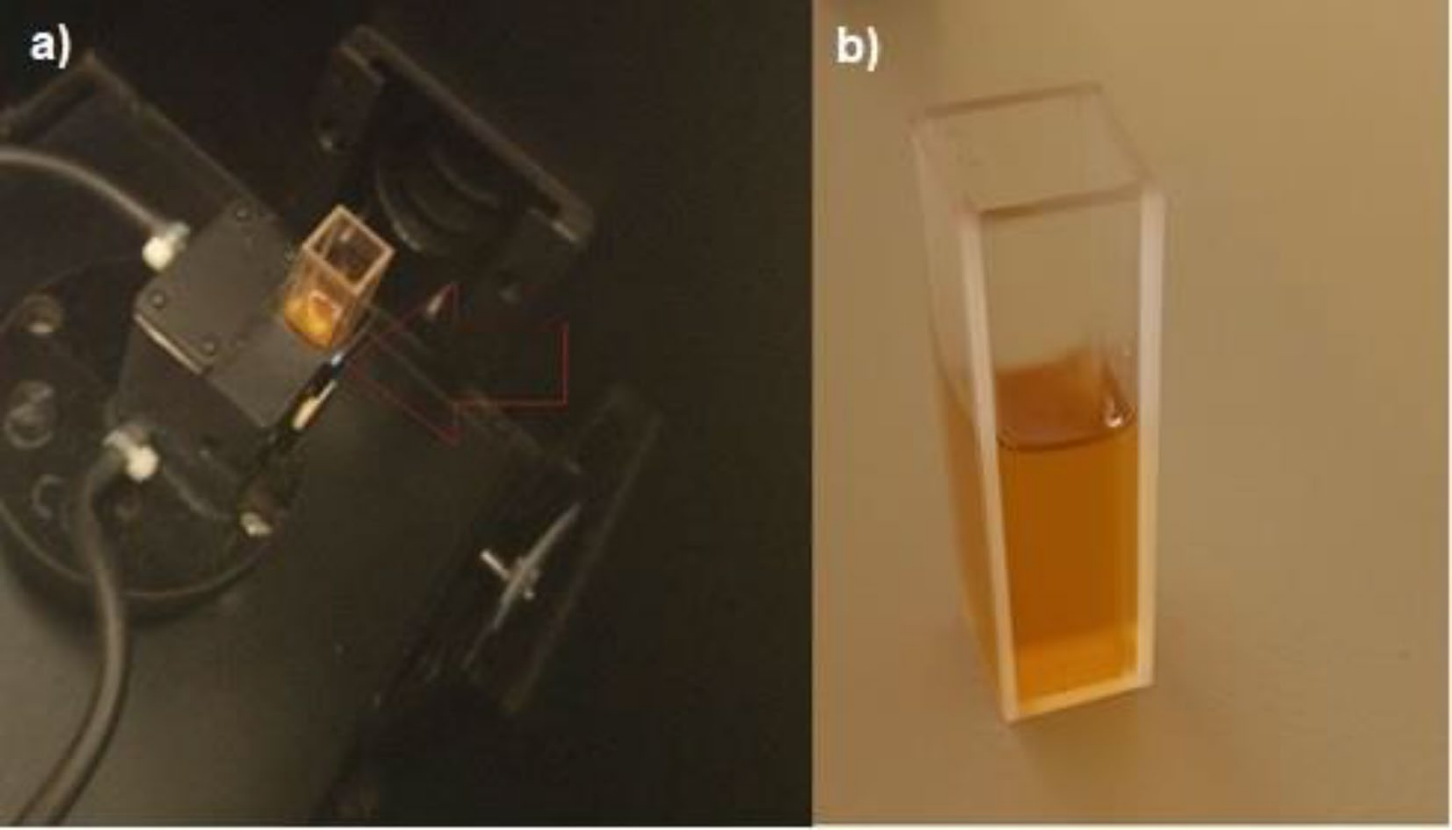
(**a**) Photo-luminescence setup and sample holder cell and, (**b**) Phendiimine molecule in ethanol solvent within an experimental quartz cell.

Figure 7 shows normalized UV–visible absorption and photo-luminescence (florescence) spectra of Phendiimine molecule in ethanol solvent. This molecule displays an intense and broad fluorescence band centered at 415 nm when the excitation wavelength is 200 nm at room temperature. The Fig. 8 shows that this compound emits blue light. There is a very small overlap between the UV–visible absorption and emission spectra. Unfortunately, there are no reports in the literature about the emission of this molecule specifically, but there are a few reports about the fluorescence of the phendione molecule. For example, the free phendione molecule (which is the starting material for the synthesis of the Phendiimine molecule) in DMF shows a very weak luminescence at approximately 543.9 nm which may be attributed to the intra molecular (π → π*) emission^36^.

### Electrochemical behavior study

**Figure 9:**
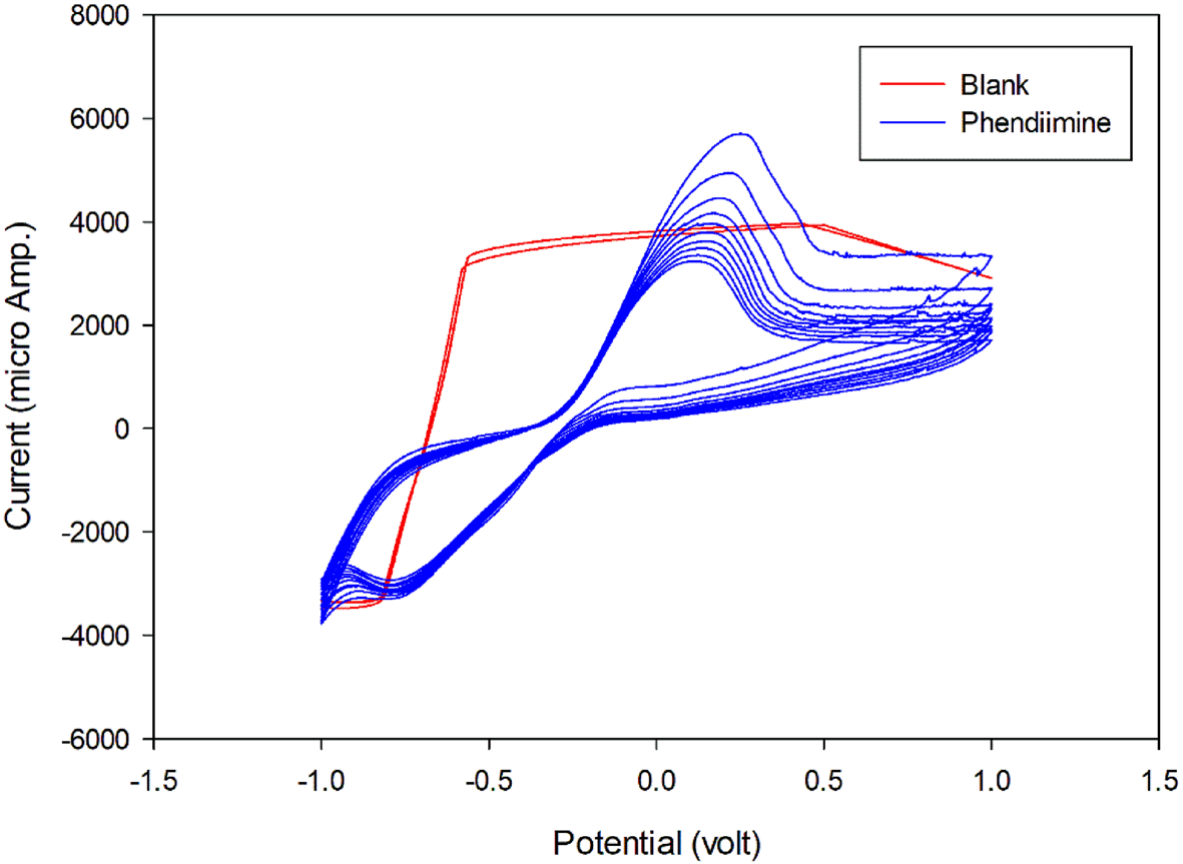
Cyclic voltammograms of Fe electrode in blank solution (red) and Phendiimine compound (1 × 10^−3^ M) in ethanol solvent and H_2_SO_4_ (1 M) as supporting electrolyte solution with Fe as working electrode with scan rate 100 mV/s (blue).

In the electrochemical experiments, a three electrode system was utilized. A platinum wire electrode served as the auxiliary electrode, while an Hg/HgO/KOH (saturated) electrode with a potential of 0.1 V against SHE electrode. A bare Fe electrode (2 mm in diameter) was used as the working electrode. Figure 9 shows, ten cycles of the cyclic voltammogram of the Phendiimine compound (1 × 10^−3^ M) in an ethanol solvent and H_2_SO_4_ (1 M) as the supporting electrolyte solution with a scan rate of 100 mV/s. It can be observed that this molecule is an electroactive material that can undergo oxidation and reduction. Phendiimine exhibits an oxide peak at + 0.2 V and a reduction peak at − 0.8 V versus reference electrode (blue colour). Although, Fe is a redox-active metal, it is not well behavior in highly corrosive H_2_SO_4_ solution. Due to the high rate of iron dissolution in acidic medium, the electrode reaction is not controlled by mass diffusion (blank as red colour). Therefore, as a result, well-defined oxidation and reduction Fe peaks cannot be observed. It appears that the reduction peak of oxygen is seen around − 0.8 V. However, this peak is not caused by oxygen, as the solution was purged with pure Argon gas for 15 min before each experiment was conducted.

### Photo sensitivity or light dependent resistance (LDR) study

**Figure 10:**
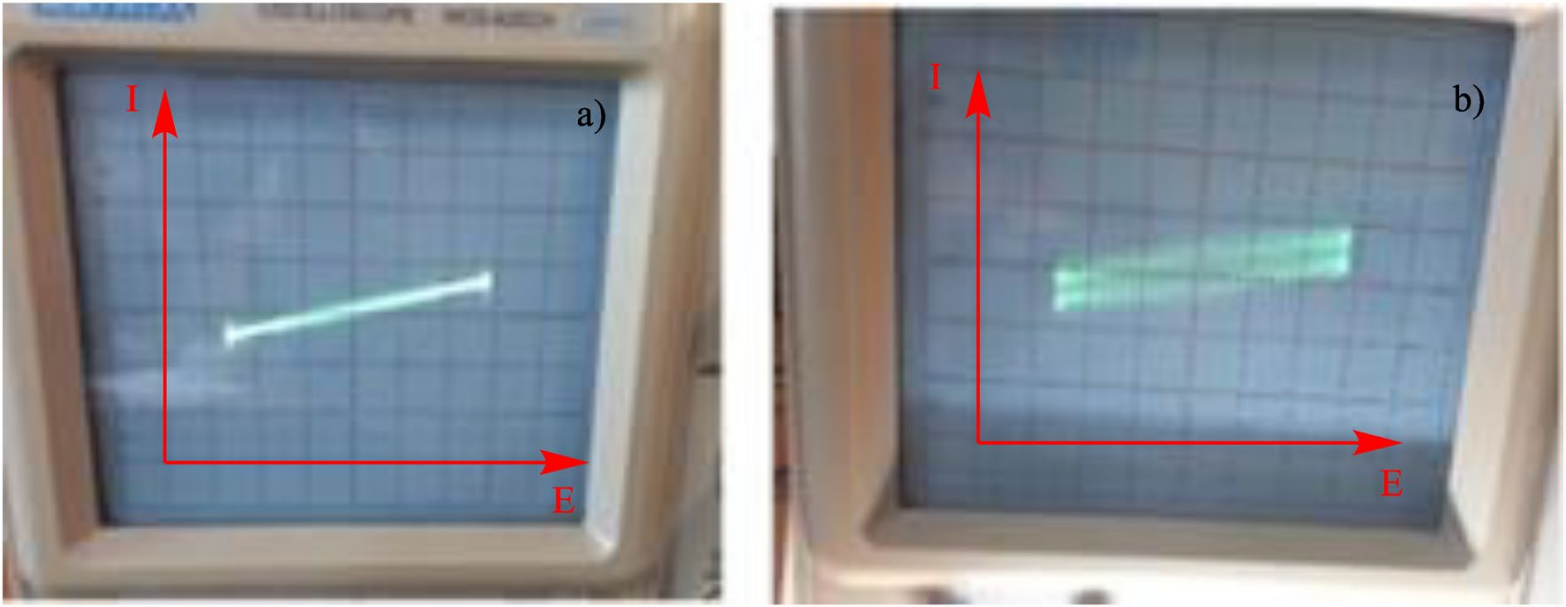
light dependent behavior of Phendiimine compound in drayed saturated KOH solution shown in oscilloscope, (**a**) in the absence of irradiation and (**b**) in the presence of irradiation.

Figure 10 show light dependent behavior of Phendiimine compound in drayed saturated KOH solution. For LDR experiments, 0.2 M of Phendiimine in 6 M of KOH aqueous solution was drayed in ambient temperature for 5 h and then it was irradiated with xenon 18 W lamp on a quartz substrate which in turn stimulated by an electrical AC sin signal with an amplitude of 5 V peak to peak and frequency of 50 Hz. This experiment shows that the i-v plot exhibits a simple resistance behavior before irradiation (Fig. 10-a) which it is doubled after irradiation (Fig. 10-b). It is clear that this compound resistance is light dependent. It is worth noting that in order to conduct LDR studies, one cannot use H_2_SO_4_ medium due to its corrosive properties and capacitive behavior, which can cause further interferences. However, using dry KOH medium does not have these issues.

**Figure 11:**
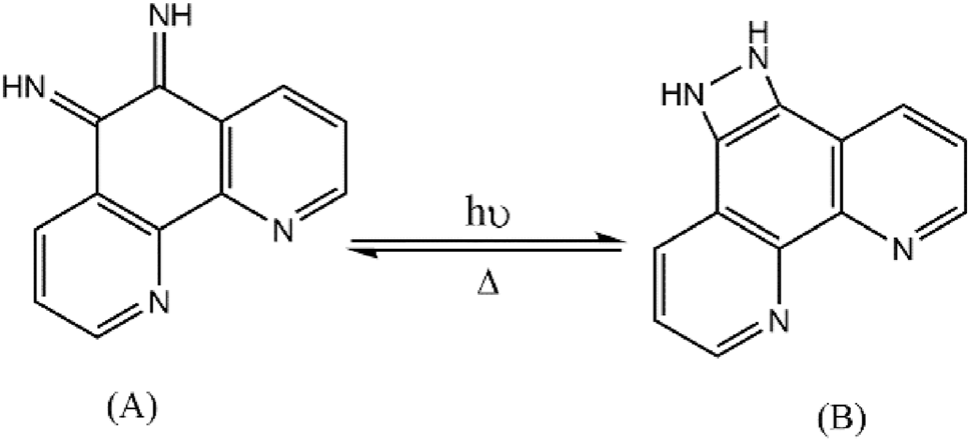
A proposed mechanism for the semiconductor behavior of Phendiimine in the presence and absence of the light.

The reason for this optical behavior in the Phendiimine molecule can be explained as a result of the interaction of light in the excitation of the electrons related to the imine double bonds of form (**A**) to create a nitrogen-nitrogen single bond for the generation of a four member fused ring (**B**), which can be seen in the Fig. 11^37–39^. Light radiation can expand and strengthen the conjugated system in the carbon skeleton structure by stimulating electrons to produce carbon–carbon double bonds and accelerate conduction. Also without the light, since the four membered cyclic ring in form (B) is unstable due to internal tension, hence, it can turn to the stable form (A) through the thermal collisions.

### Hot point probe test

**Figure 12:**
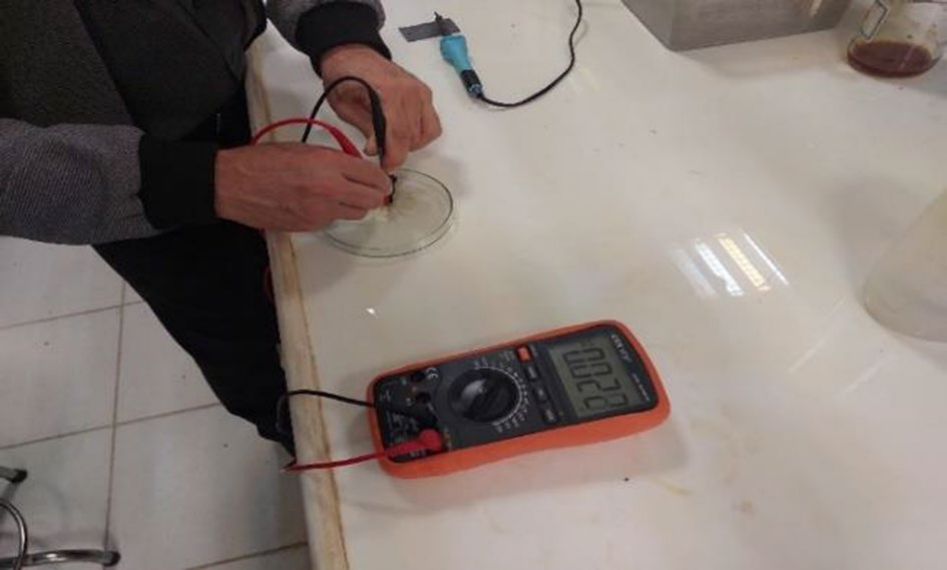
Hot point test of Phendiimine molecule thin film.

This is a simple, accurate and quick test for determining whether a semiconductor sample (or its thin film) is n-type or p-type^40–42^. In this test, the hot probe is connected to the positive terminal of the multi-meter while the cold probe of the multi-meter is connected to the negative terminal with a distance of approximately 1 cm from the hot probe. When the cold and hot probes are applied to an n-type semiconductor thin film, a positive voltage readout is obtained in the multi-meter. Conversely, whereas for a p-type semiconductor, a negative voltage is obtained. As shown in Fig. 12, the Phendiimine thin film exhibits a positive voltage of 0.022 V, indicating that it is an n-type semiconductor.

### Tauc plot

A Tauc plot based on the following equation is used to determine the optical bandgap value of either amorphous or disordered semiconductors.

$$\left( {\alpha \cdot h\upsilon } \right)^{{{1}/\gamma }} = {\text{B}}\left( {h\upsilon - E_{{\text{g}}} } \right)$$

For a γ factor equal to 0.5 viz. or 1/γ = 2, it denotes a direct band gap semiconductor. For a γ factor equal to 2 viz. or 1/γ = 0.5, it denotes an indirect band gap semiconductor. Here, α represents the absorption coefficient and B is a constant^42,43^.

**Figure 13:**
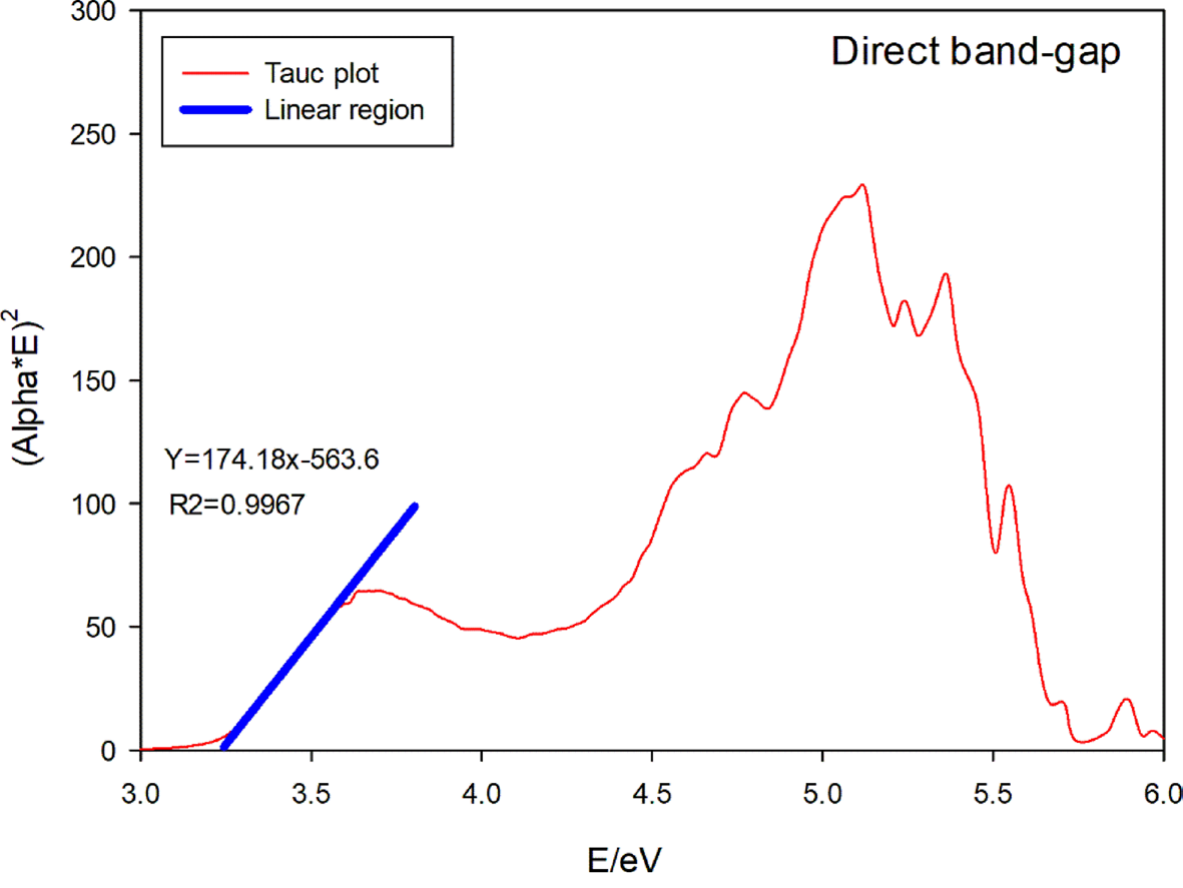
Tauc plot for direct band-gap along with its linear region.

**Figure 14:**
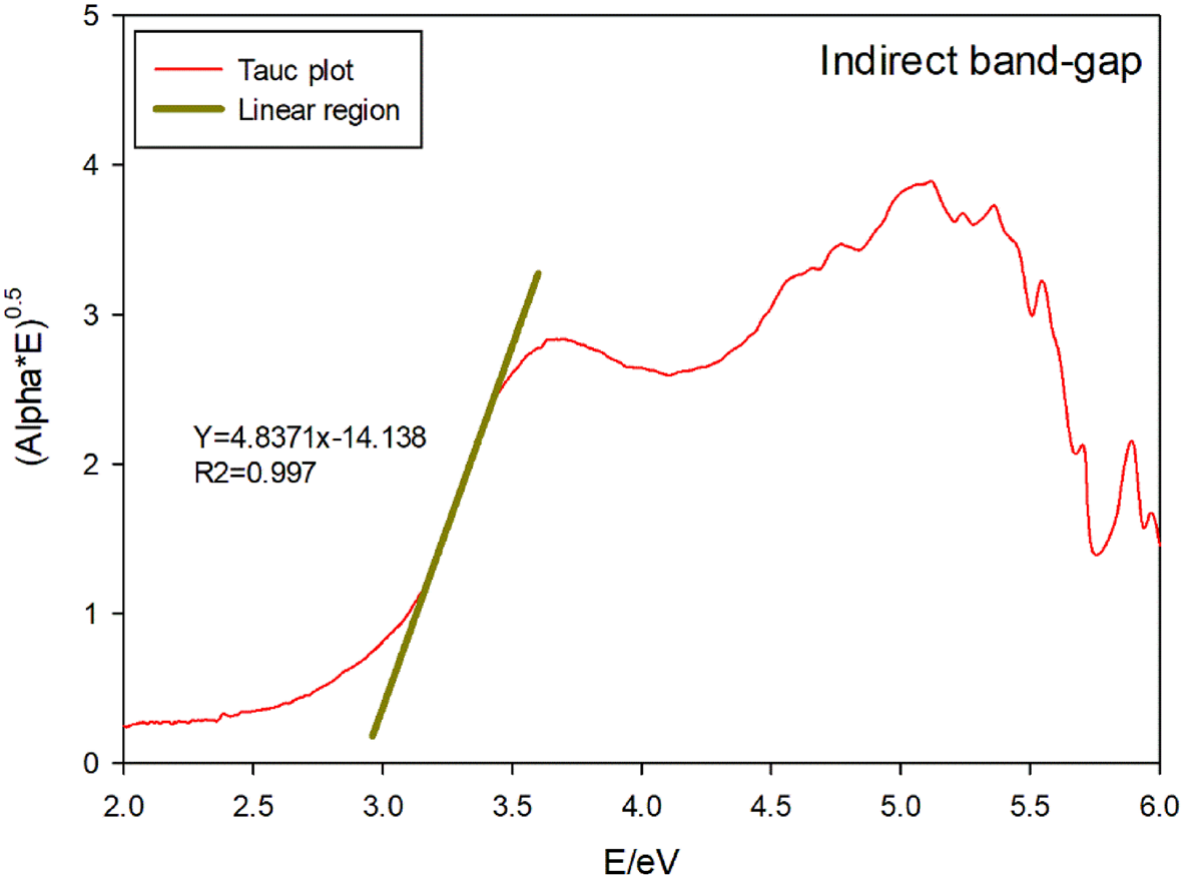
Tauc plot for indirect band-gap along with its linear region.

Figure 13 shows the Tauc plot for direct band-gap of Phendiimine molecule along with its linear region of this plot. As can be seen from the fitted linear region, the direct band gap is 3.23 eV. Figure 14 shows the Tauc plot for indirect band-gap of Phendiimine molecule along with its linear region of this plot the linear region of this plot. As can be seen from the fitted linear region, the indirect band gap is 2.92 eV. By comparing Figs. 13 and 14, it is clear that the linear region is pronounced in Fig. 13. Therefore, it seems that Phendiimine molecule is a direct band-gap semiconductor.

## Experimental

The melting point of synthesized compound **3** (1,10-phenanthroline-5,6-diimine) was measured using the Barnstead Electrothermal 9200 apparatus and the IR spectrum was obtained using the Thermo-Nicolet Nexus 670 FT-IR spectrometer. Additionally, the ^1^H and ^13^CNMR spectra for compound **3** were recorded on a BRUKER DRX-250 AVANCE instrument using DMSO-d_6_ as the solvent and TMS as the internal standard at 250 MHz. 1,10-phenanthroline-5,6-dione was synthesized according to the previously reported procedure^13,24,44^. The chemicals 2-Picolylamine, Sulfuric acid and solvents were purchased from Merck and Sigma-Aldrich companies and used without further purification.

## General procedure

The mixture of 1,10-phenanthroline-,6-dione (phendione) (0.22 g, 2 mmol) was dissolved in 20 mL of ethanol and was then added 2-Picolylamine (0.43 g, 4 mmol) in 10 mL EtOH at room temperature. Then 2 drops of concentrated sulfuric acid were added to the reaction medium, slowly. The reaction mixture was stirred under reflux condition for 1.5 h. After cooling to room temperature, ethanol was removed by slow evaporation manner. After removal of solvent the crude product were washed with cold diethyl ether (2 × 3 mL) to give the corresponding product as greenish-yellow solid.

Yield: 94%, 0.21 g. m.p. 273 °C (decomposed). IR (KBr) (υ_max_, cm^−1^): 3440 (NH) and 1570 (C = N). ^1^H NMR (250 MHz, DMSO-d_6_): δ 7.15 (dd, *J*_1_ = 8.0,* J*_2_ = 4.0 Hz, 2H, CH), 8.57 (d, *J* = 8.0 Hz, 2H, CH), 8.00 (d, *J* = 4.0 Hz, 2H, CH), 9.47 (br s, 2H, NH).

## Conclusion

Here, one facile method for direct synthesis of 1,10-Phenanthroline-5,6-diimine (Phendiimine) compound introduced. This molecule showed solvatochromic behavior in two solvents and also showed photo-sensitivity against visible light, together with photoluminescence behavior in both water and ethanol. Finally, it showed electrochemical redox activity with Fe electrode in ethanol and H_2_SO_4_ solution. Based on our findings, it appears that the Phendiimine molecule has an impact on various fields such as medicine, the construction of photovoltaic cells with high yield, photo-controllers, miniaturized real photostimulated motors, photoswitches, electronic light-dependent resistance (LDR) elements, singlet fission materials in solar cells and more.

### Supplementary Information

Supplementary Information.

## Data Availability

All data generated or analysed during this study are included in this published article [and its supplementary information files].

## References

[1] Berná J, Leigh DA, Lubomska M, Mendoza SM, Perez EM, Rudolf P, Teobaldi G, Zerbetto F (2005). Macroscopic transport by synthetic molecular machines. Nat. Mat..

[2] Briane V, Vimond M, Kerverann C (2020). An overview of diffusion models for intracellular dynamics analysis. Brief. Bioinf..

[3] Maćkała A, Magdziarz M (2019). Statistical analysis of superstatistical fractional Brownian motion and applications. Phyz. Rev. E..

[4] Silva GT, Bruus H (2014). Acoustic interaction forces between small particles in an ideal fluid. Phyz. Rev. E..

[5] Sahu ID, McCarrick RM, Lorigan GA (2013). Use of electron paramagnetic resonance to solve biochemical problems. Bio. Chem..

[6] Suda M, Thathong Y, Promarak V, Kojima H, Nakamura M, Shiraogawa T, Ehara M, Yamamoto HM (2019). Light-driven molecular switch for reconfigurable spin filtersamamoto. Nat. Commun..

[7] Yi SJ, Kim M, Kim D, Kim HD, Kim KC (2016). Transient temperature field and heat transfer measurement of oblique jet impingement by thermographic phosphor. Int. J. Heat Mass. Transfer..

[8] Chen Y, Liu Y, Lu S, Ye S, Gu H, Qiang J, Li Y, Chen X (2020). Photostimulated spiropyran for instantaneous visualization of thermal field distribution and flow pattern. J. Am. Chem. Soc..

[9] Rezvani AR, Saravani H, Hadadzadeh H (2010). Synthesis, crystal structure, electrochemical and fluorescence studies of a novel Zn (II)-fluorophore, 1, 10-phenanthroline-5, 6-dione (phen-dione). J. Iran. Chem. Soc..

[10] Fletcher NC, Robinson TC, Behrendt A, Jeffery JC, Reeves ZR, Ward MD (1999). Structural, electrochemical and UV/VIS/NIR spectroelectrochemical properties of diastereomerically pure dinuclear ruthenium complexes based on the bridging ligand phenanthroline-5,6-diimine, and a mononuclear by-product with a peripheral isoimidazole group. J. Chem. Soc. Dalton. Trans..

[11] Liu Y, Duan ZU, Zhang HY, Jiang XL, Han JR (2005). Selective binding and inverse fluorescent behavior of magnesium ion by podand possessing plural imidazo[4,5-*f*]-1,10-phenanthroline groups and its Ru(II) complex. J. Org. Chem..

[12] Zhang Y, Hu F, Wang B, Zhang X, Liu C (2015). Enantioselective recognition of chiral carboxylic acids by a β-amino acid and 1,10-phenanthroline based chiral fluorescent sensor. Sensors.

[13] Bodige S, MacDonnell FM (1997). Synthesis of free and ruthenium coordinated 5,6-diamino-1,10-phenanthroline. Tetrahedron Lett..

[14] Campagna S, Serroni S, Bodige S, MacDonnell FM (1999). Absorption spectra, photophysical properties, and redox behavior of stereochemically pure dendritic ruthenium(II) tetramers and related dinuclear and mononuclear complexes. Inorg. Chem..

[15] Starikova AA, Minkin VI (2018). Adducts of transition metal complexes with redox-active ligands: The structure and spin-state-switching rearrangements. Russ. Chem. Rev..

[16] Abbasi A, Geranmayeh S, Saniee V, Juibari NM, Badiei AZ (2012). Synthesis and characterization of tetrapyridophenazine ligand and its novel 1-D metal-organic wave-like coordination polymer of Ni (II) ion. Kristallogr..

[17] Schilt AA (1969). Analytical Applications of 1,10-Phenanthroline and Related Compounds.

[18] Kao HC, Hsu CJ, Hsu CW, Lin CH, Wang WJ (2010). Aza-bridged bis-1, 10-phenanthroline acyclic derivatives: Synthesis, structure, and regioselective alkylation. Tetrahedron Lett..

[19] Krapcho AP, Sparapani S, Leenstra A, Seitz JD (2009). Displacement reactions of 2-chloro- and 2,9-dichloro-1,10-phenanthroline: Synthesis of a sulfur-bridged bis-1,10-phenanthroline macrocycle and a 2,2′-amino-substituted-bis-1,10-phenanthroline. Tetrahedron Lett..

[20] Nam H, Jeong M, Sohn OJ, Rhee JI, Oh J, Kim Y, Lee S (2007). Synthesis of phenanthroline derivative by Suzuki coupling reaction and the use of its ruthenium complex as an optical pH sensor. Inorg. Chem. Commun..

[21] Sammes PG, Yahioglu G (1994). 1,10-Phenanthroline: A versatile ligand. Chem. Soc. Rev..

[22] Maghsoodlou MT, Marandi G, Hazeri N, Aminkhani A, Kabiri R (2007). A facile synthesis of oxazolo[3,2-*a*][1,10]phenanthrolines via a new multicomponent reaction. Tetrahedron Lett..

[23] Maghsoodlou MT, Habibi Khorassani SM, Saghatforoush L, Maghfuri F, Marandi G, Kabiri R (2008). Stereoselective synthesis of helical dihydrodipyrrolophenanthroline and Hindrance Hexa *tert*-butyl carboxylatodipyrrolophenanthroline from reaction between 1,10- phenanthroline and dialkyl acetylenedicarboxylates. J. Heterocycl. Chem..

[24] Maghsoodlou MT, Habibi Khorassani SM, Hazeri N, Heydari R, Marandi G, Nassiri M (2006). The new γ-spiroiminolactone synthesis by reaction between alkyl or aryl isocyanides and 1,10-phenanthroline-5,6-dione in the presence of acetylenic esters. J. Chem. Res..

[25] Marandi G, Hazeri N, Maghsoodlou MT, Habibi Khorassani SM, Akbarzadeh Torbati N, Rostami-Charati F, Skelton BW, Makha M (2013). Synthesis of cyano-pyrrolo[1,2-*a*][1,10]phenanthroline derivatives using a multicomponent condensation. J. Heterocycl. Chem..

[26] Hassanzadeh A, Loghmani-Khouzani H, Sadeghi MM, Mehrabi H (2007). Solvatochromic, spectroscopic and DFT studies of a novel synthesized dye: l-(4-Dimethylaminophenyl)-2-(5*H*-phenanthridine-6-ylidene)-ethanone (6-KMPT). Spectrochim. Acta Part A..

[27] Starikova AA, Chegerev MG, Starikov AG, Minkin VI (2019). Rational design of electronically labile dinuclear Fe and Co complexes with 1,10-phenanthroline-5,6-diimine: A DFT study. J. Comput. Chem..

[28] Ghumaan S, Sarkar B, Patra S, Slageren JV, Fiedler J, Kaim W, Lahiri GK (2005). Sensitive oxidation state ambivalence in unsymmetrical three-center (M/Q/M) systems [(acac)_2_Ru(μ-Q)Ru(acac)_2_]^*n*^, Q = 1,10-phenanthroline-5,6-dione or 1,10-phenanthroline-5,6-diimine (*n* = +, 0, −, 2−). Inorg. Chem..

[29] MacDonnell FM, Bodige S (1996). Efficient stereospecific syntheses of chiral ruthenium dimers. Inorg. Chem..

[30] Torres AS, Maloney DJ, Tate D, Kinsel GR, Walker AK, MacDonnell FM (1997). First-generation chiral metallodendrimers: Stereoselective synthesis of rigid *D*_3_-symmetric tetranuclear ruthenium complexes. J. Am. Chem. Soc..

[31] Iino R, Kinbara K, Bryant Z (2020). Introduction: Molecular motors. Chem. Rev..

[32] Pooler DRS, Lubbe AS, Crespi S, Feringa BL (2021). Designing light-driven rotary molecular motors. Chem. Sci..

[33] Kistemaker JCM, Lubbe AS, Feringa BL (2021). Exploring molecular motors. Mater. Chem. Front..

[34] Mich J, Sykes ECH (2009). Molecular rotors and motors: Recent advances and future challenges. ACS Nano.

[35] Regen-Pregizer BL, Ozcelik A, Mayer P, Hampel F, Dube H (2023). A photochemical method to evidence directional molecular motions. Nat. Commun..

[36] Boghaei DM, Behzadian Asl F (2007). Synthesis, characterization and fluorescence spectra of mixed ligand Zn(II), Cd(II) and Hg(II) complexes with 1,10-phenanthroline-5,6-dione ligand. J. Coord. Chem..

[37] Prasad SK (2012). Photostimulated and photosuppressed phase transitions in liquid crystals. Angew. Chem. Int. Ed..

[38] Cai S, Deng W, Huang F, Chen L, Tang C, He W, Long S, Li R, Tan Z, Liu J, Shi J, Liu Z, Xiao Z, Zhang D, Hong W (2019). Light-driven reversible intermolecular proton transfer at single molecule junctions. Angew. Chem. Int. Ed..

[39] Terao F, Morimoto M, Irie M (2012). Light‐driven molecular‐crystal actuators: rapid and reversible bending of rodlike mixed crystals of diarylethene derivatives. Angew. Chem. Int. Ed..

[40] Morab S, Sundaram MM, Pivirikas A (2023). Review on charge carrier transport in inorganic and organic semiconductors. Coatings.

[41] Sawatzki-Park M, Wang SJ, Kleemann H, Leo K (2023). Highly ordered small molecule organic semiconductor thin-films enabling complex high-performance multi-junction devices. Chem. Rev..

[42] Tariq GH, Asghar G, Shifa MS, Anis-Ur-Rehman M, Ullah S, Shah ZA, Ziani I, Tawfeek AM, Sher A (2023). Effect of copper doping on plasmonic nanofilms for high performance photovoltaic energy applications. Phys. Chem. Chem. Phys..

[43] Makuła P, Pacia M, Macyk W (2018). How to correctly determine the band gap energy of modified semiconductor photocatalysts based on UV−Vis spectra. J. Phys. Chem. Lett..

[44] Yamada M, Tanaka Y, Yoshimoto Y, Kuroda S, Shimao I (1992). Synthesis and properties of diamino-substituted dipyrido (3, 2-a: 2′, 3′-c) phenazine. Bull. Chem. Soc. Jpn..

