# Supplementary material for: The rapid synthesis of 1,10-phenanthroline-5,6-diimine (Phendiimine) and its fascinating photo-stimulated behavior
Source: Sci Rep. 2024 Apr 11;14:8464. doi: 10.1038/s41598-024-59272-4 (PMC11009400; doi:10.1038/s41598-024-59272-4)

Supporting Information of

The rapid synthesis of 1,10-phenanthroline-5,6-diimine and its fascinating photo-stimulated behavior

Ghasem Marandi,[1,*] Ali Hassanzadeh[2]

[1] Department of Organic Chemistry, Faculty of Chemistry, Urmia University, Urmia, Iran

[2] Department of Physical Chemistry, Faculty of Chemistry, Urmia University, Urmia, Iran

Table of Contents

1- **General Information**
2- **Experimental Procedures**
3- **References**
4- **IR spectrum of the Product**
5- **^{1}H NMR Spectrum of the Product**
6- **^{13}C NMR Spectrum of the Product**
7- **The UV-Visible spectra of Phendiimine**
8- **Photo-luminescence spectra of Phendiimine**
9- **Cyclic voltammograms of Phendiimine and Fe electrode**
10- **Tauc plot for direct band-gap along with its linear region**
11- **Tauc plot for indirect band-gap along with its linear region**

1- General information

The melting point of synthesized compound **3** (1,10-phenanthroline-5,6-diimine) were measured on Barnstead Electrothermal 9200 apparatus and the IR spectrum was performed on Thermo-Nicolet Nexus 670 FT-IR spectrometer, respectively. Also, the ^{1}H and ^{13}CNMR spectra for the compound **3** was recorded on a BRUKER DRX-250 AVANCE instrument using DMSO-d_6 as a solvent and TMS as internal standard at 250 MHz. 1,10-phenanthroline-5,6-dione was synthesized according to the previously reported procedure.[1-3] 2-Picolylamine, Sulfuric acid and solvents were purchased from Merck and Sigma-Aldrich companies and used without further purification.

2- Experimental procedures

The mixture of 1,10-phenanthroline-,6-dione (phendione) (0.22 g, 2 mmol) was dissolved in 20 mL of ethanol and was then added 2-Picolylamine (0.43 g, 4 mmol) in 10 mL EtOH at room temperature. Then 2 drops of concentrated sulfuric acid were added to the reaction medium, slowly. The reaction mixture was stirred under reflux condition for 1.5 h. After cooling to room temperature, ethanol was removed by slow evaporation manner. After removal of solvent the crude product were washed with cold diethyl ether (2×3 mL) to give the corresponding product as greenish-yellow solid.

Yield: 94%, 0.21 g. m.p. 273 °C (decomposed). IR (KBr) (υ_{max}, cm^{-1}): 3440 (NH) and 1570 (C=N). ^{1}H NMR (250 MHz, DMSO-d_6): δ 7.15 (dd, J_1 = 8.0, J_2 = 4.0 Hz, 2H, CH), 8.57 (d, J = 8.0 Hz, 2H, CH), 8.00 (d, J = 4.0 Hz, 2H, CH), 9.47 (br s, 2H, NH).

3- References

(1) Bodig, S.; MacDonnell, F.M. *Tetrahedron Lett.*, **1997**, *38*, 8159-8160.
(2) Yamada, M.; Tanaka, Y.; Yoshimoto, Y.; Kuroda, S.; Shimao, I. *Bull. Chem. Soc. Jpn.* **1992**, *65*, 1006-1011.
(3) Maghsoodlou, M.T.; Habibi-Khorassani, S.M.; Hazeri, N.; Heydari, R.; Marandi, G.; Nassiri, M. *J. Chem. Res.* **2006**, 225-227.

4- The IR spectrum of phendiimine

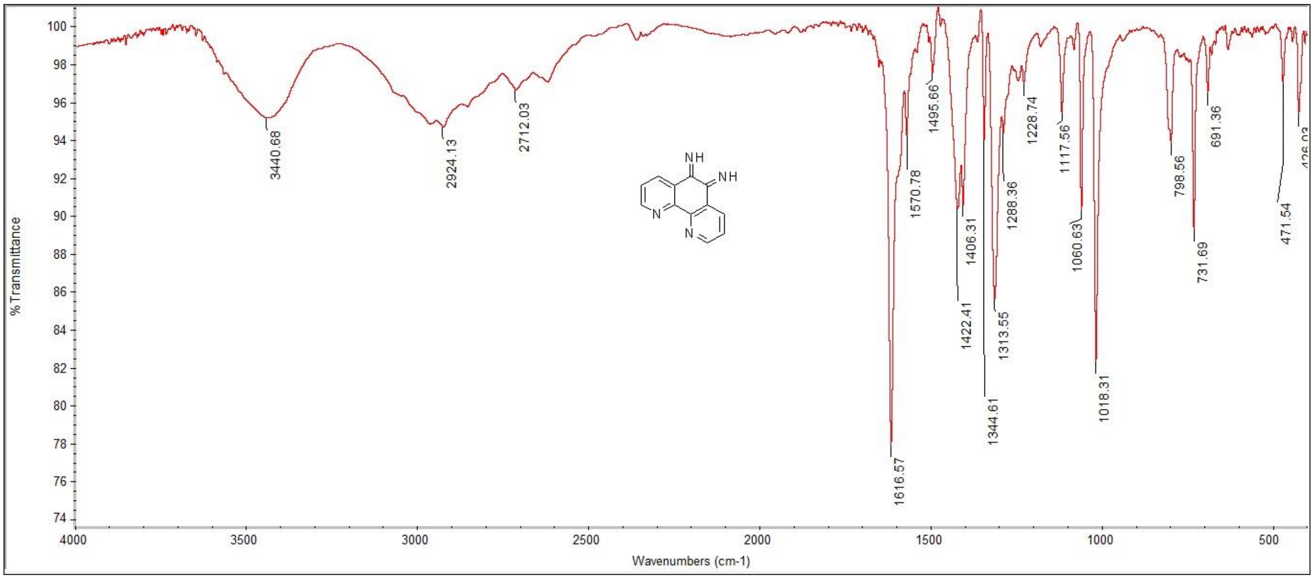

5- The ^{1}H NMR spectrum of phendiimine

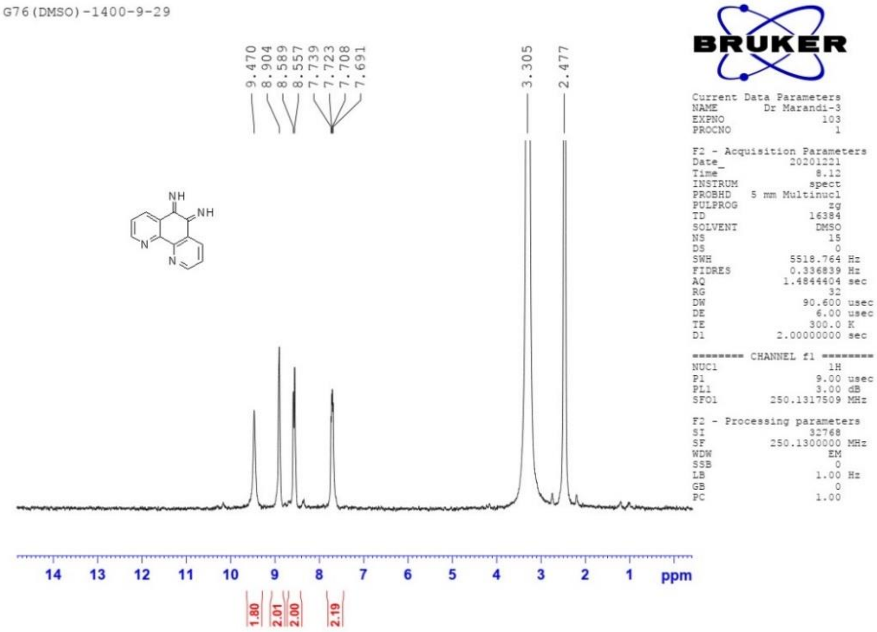

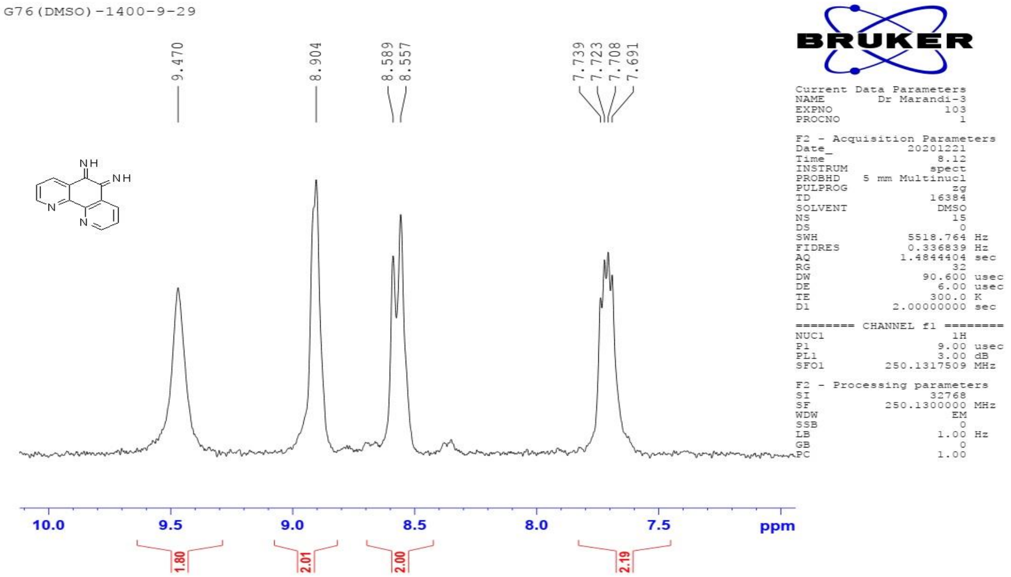

6- The ^{13}C NMR spectrum of phendiimine

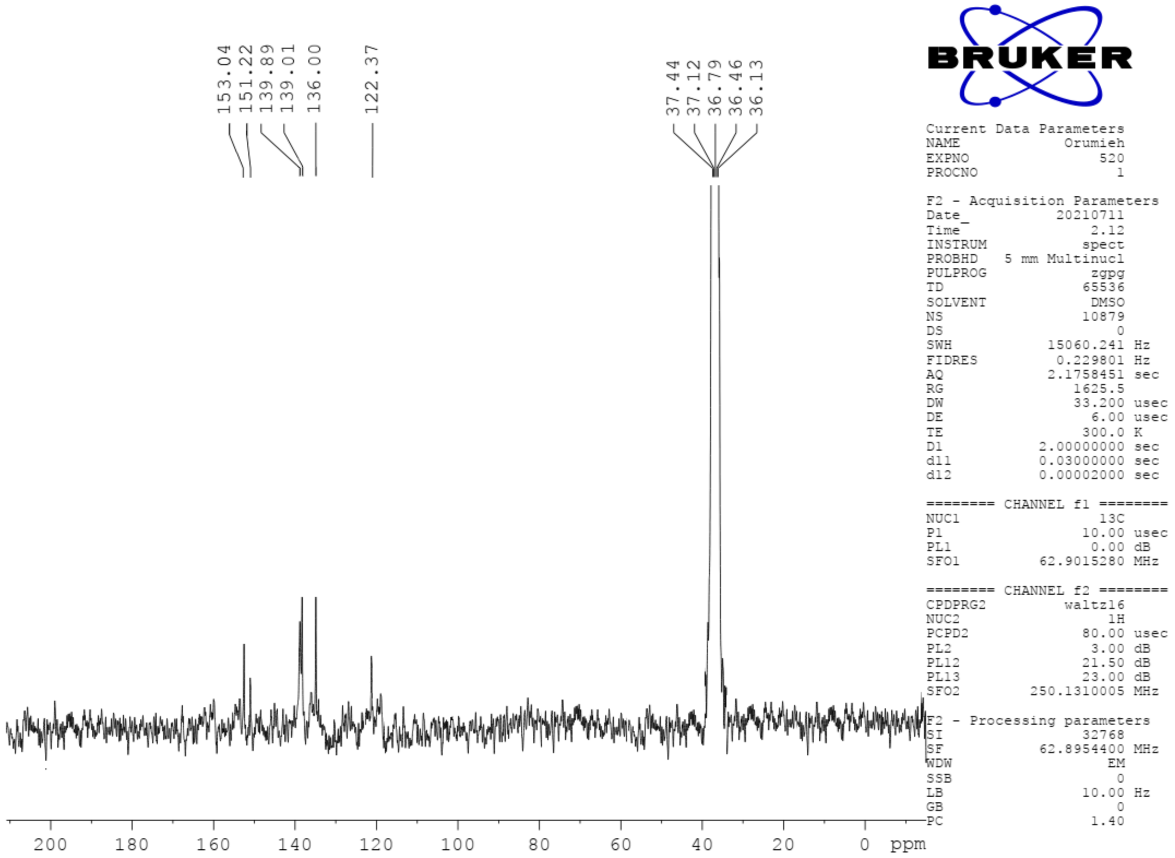

7- The UV-Visible spectra of Phendiimine molecule in ethanol and water solvents.

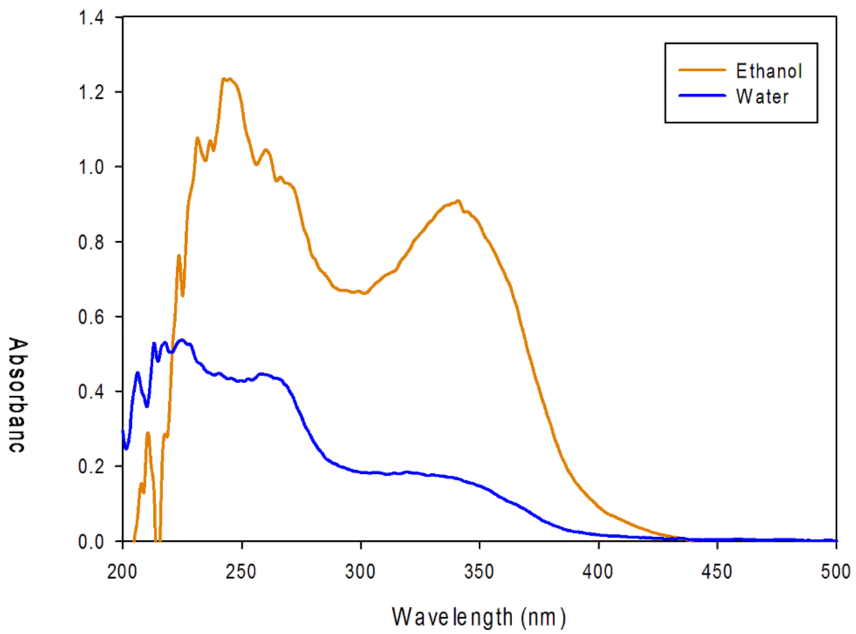

8- Photo-luminescence spectra of Phendiimine molecule in ethanol solvent; Excitation wavelength =200 nm.

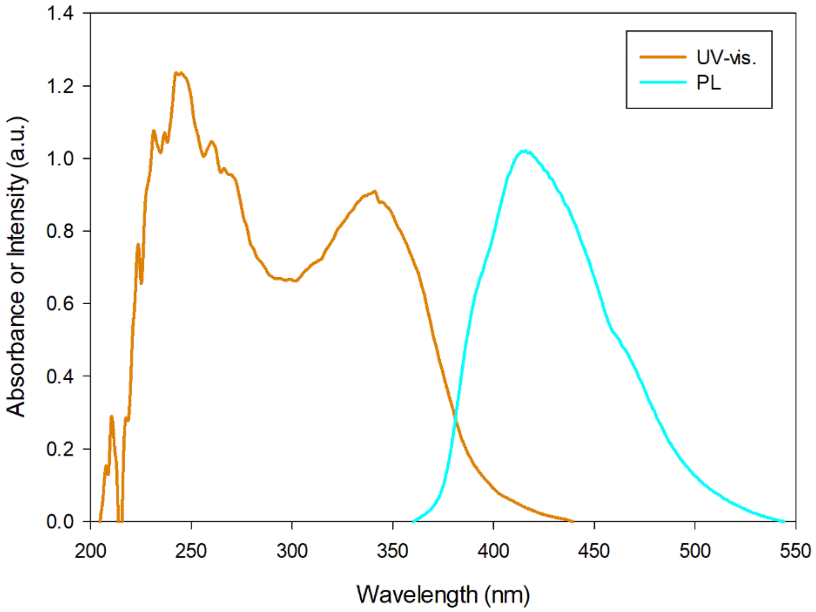

9- Cyclic voltammograms of Fe electrode in blank solution (red) and Phendiimine compound (1×10^{-3} M) in ethanol solvent and H_2SO_4 (1 M) as supporting electrolyte solution with Fe as working electrode with scan rate 100 mV/s (blue).

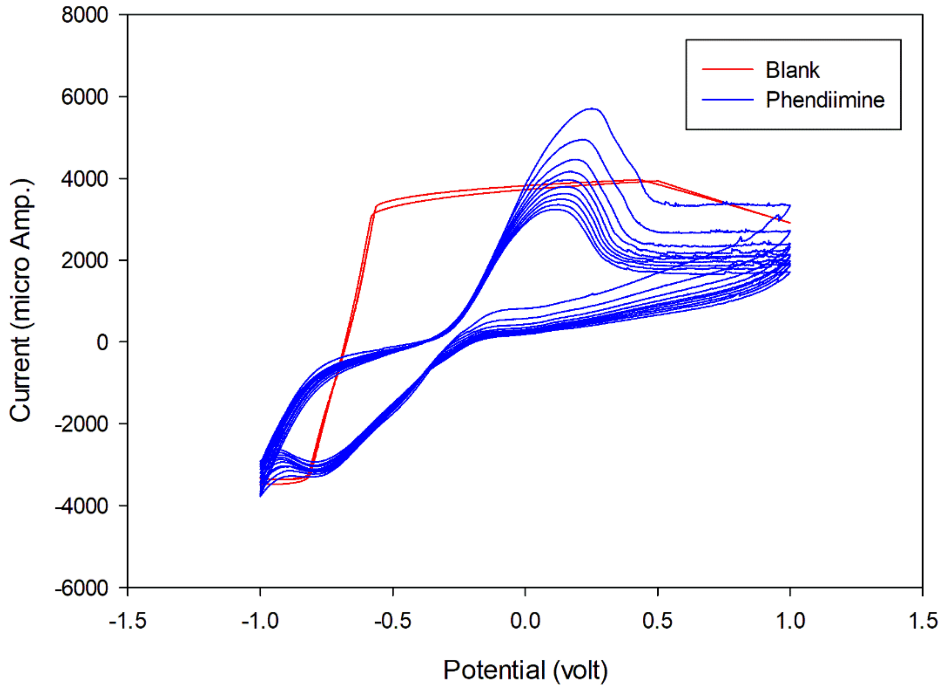

10- Tauc plot for direct band-gap along with its linear region.

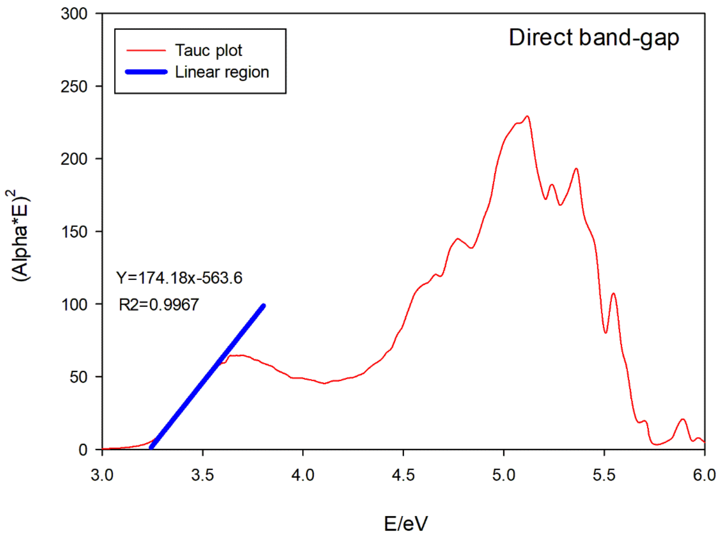

11- Tauc plot for indirect band-gap along with its linear region.

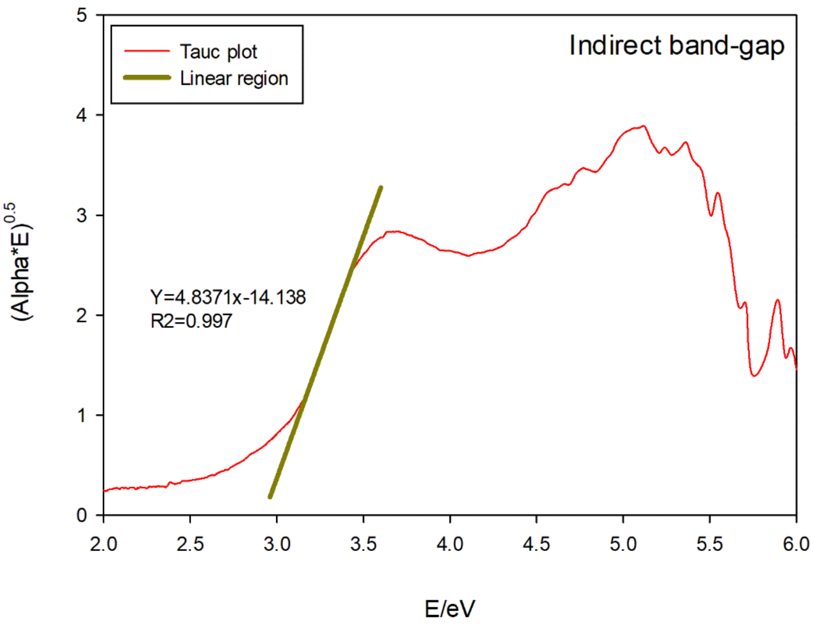

Supplement: Supplementary file 1 — Supplementary Information. [file 41598_2024_59272_MOESM1_ESM.pdf]
